# Factors of Parental Preparation of Children with Mental Illnesses for Their Independent Living after Their Own Death

**DOI:** 10.3390/healthcare10122360

**Published:** 2022-11-24

**Authors:** Kyoko Yoshioka-Maeda, Hitoshi Fujii, Masako Kageyama, Soichi Takamura

**Affiliations:** 1 Department of Community Health Nursing, Division of Health Sciences and Nursing, Graduate School of Medicine, The University of Tokyo, Tokyo 113-0033, Japan; 2 Department of Medical Statistics, School of Nursing, Mejiro University, Saitama 339-8501, Japan; 3 Division of Health Sciences, Graduate School of Medicine, Osaka University, Osaka 565-0871, Japan; 4 Department of Mental Health Nursing, Graduate School of Nursing, University of Shizuoka, Shizuoka 422-8526, Japan

**Keywords:** community, death preparation, independent living, mental illness, parent, recovery

## Abstract

Families of children with mental illnesses are often concerned about living in the community after their parents’ death. The cross-sectional study aimed to examine the association between how parents prepare adult children with mental illnesses to live independently after the death of the parent(s). The participants were 1112 members of 46 family support groups for mental illnesses in Tokyo, Japan. The age of the people with mental illness was 40s, and that of their parents was 70s. Logistic regression analysis showed that mothers’ support in daily living, no income or pension for disabled people, staying at home during free time, and parental livelihood being the same as the person with mental illness were factors that were negatively associated with the independent living of people with mental illness. In contrast, parental participation in the family group and creating a system for securing regular living expenses of the person with mental illness were positively associated with independent living. The results suggest that parents need to promote their children’s recovery and prepare them financially by forecasting their independent living after their own death.

## 1. Introduction

In light of the global promotion of deinstitutionalization, the place of living for the mentally disabled is shifting to the community [1]. Stigma against people with mental illness is associated with seeking and using mental health services and their recovery [2]. Japanese people have strong stigmas regarding mental illness [3] and express a negative attitude toward developing mental healthcare facilities in their community [4]. This is one reason Japan is facing difficulties in reducing the number of psychiatric beds and inpatients [5]. Additionally, outreach and individual care services for people with mental illnesses still lag behind other developed countries [6,7]. Therefore, approximately 70% of people with mental illnesses under 65 years old live with their parents [8].

Caregiver burden is a major issue for families who have child(ren) with mental illness [9,10]. To compensate for the lack of social resources for people with mental illnesses, their parents provide them with care [11]. Parents invest their time, energy, and money over the long term because of their children’s unemployment and unmarried status [12]. Because of the Japanese culture of shame and *sekentei* (social appearance), parents do not want to seek help and try to manage their children’s health and life-related issues by themselves [13]. Therefore, families who have children with mental illnesses have concerns about living in the community after the parents’ death [14].

Generally, older people accept their death and gradually prepare for it [15]. In cases of inadequate preparation for the death of parents, the grief experienced by a person with mental illness is aggravated and prolonged [16]. Because parental death impacts children’s mental health, parents with a life-limiting illness often communicate with their children to prepare them for their own death [17]. Previous studies have focused on communicating with older adults regarding their advanced care planning [18,19,20,21], parental death preparation for children with cancer or other serious diseases [22,23], and mental illnesses [24]. A literature review also revealed that support programs for parentally bereaved children aged 0–18 years effectively prevented mental health issues [23]. However, only one qualitative study revealed that older parents who have adult children with mental illness prepared for the children’s independent living after their own death, and factors related to their preparations for their children remain unclear [24]. Japan is a super-aged society; the 8050 issue has become a major social problem, where older parents living on pensions are increasingly facing problems taking care of their children in their 40s and 50s [25]. Identifying these factors will enhance their independence and social participation in recovery.

Therefore, this study aimed to examine the association of how parents prepare their children with mental illnesses for independent living after the death of a parent.

## 2. Materials and Methods

### 2.1. Design and Setting

This study used a cross-sectional study design. It was conducted from 10 November to 31 December 2021. The study participants were members of the 46 family support groups for mental illness in Tokyo, Japan. Each group had a different number of members, which was not disclosed. Therefore, we needed to identify the total number of potential study participants through the presidents of the family support group. The principal researcher explained the study’s aim and methods to the presidents of each family support group at the board’s general meeting on 5 November 2021. Additionally, the principal researcher asked them whether they had the intention to participate in this study or not. If they intended to participate in this study, the presidents of the family group responded with the total number of members.

Among the 46 groups, 30 presidents responded and expressed their intention to participate in this study. The total number of their members was 1112. We sent anonymous questionnaires to the 30 groups. To protect the privacy of the group members, each family group sent a questionnaire to their members.

### 2.2. Ethical Considerations

The Institutional Review Board of the principal researcher’s affiliated institution approved the study, which was based on the principles of the Declaration of Helsinki (September 15, 2021; No. NIPH—IBRA#12339). The study objectives and methods were explained to the participants in writing. Informed consent was obtained from all study participants before returning completed questionnaires.

### 2.3. Measures

We developed a questionnaire based on previous studies [9,10,11,14] and discussions with 12 board members of the Tokyo Family Support Group Association for mental illness.

#### 2.3.1. Demographic Data of People with Mental Illnesses

The questionnaire included demographic data of the people with mental illnesses: age, sex, educational background, diagnosis, number of years since the onset of mental illness, independent management of medication, cohabitants, living place, support in daily living, income, and how to spend their free time. We also enquired about instrumental activities of daily living (IADL) [26] of children with mental illnesses; for example, preparing meals, housing chores, financial management, taking medication, making telephone calls, shopping for groceries, and using public transportation, scores ranging from 0 to 5 [0 (independent), 1 (sometimes conducted with the assistance), 2 (always assisted and implemented), 3 (all implemented by others), 4 (not implemented at all), and 5 (unknown)].

#### 2.3.2. Demographic Data of the Parents

The questionnaire included parents’ age, relationships, educational background, household income, whether their livelihood was the same as the person with mental illness, the presence of disease under treatment, and participation in the family support group.

#### 2.3.3. Family Stigma

We assessed family stigma as follows: “having a family member with mental illness makes my family feel ashamed” and “having a family member with mental illness makes me feel ashamed” [27]. The total score ranged from 2 to 10, using a five-point Likert scale (1 = never to 5 = always). Higher scores indicated a greater degree of family stigma. Cronbach’s alpha for this subscale was 0.949.

#### 2.3.4. Parental Preparations for Their Death and the Independent Living of Their Children with Mental Illness after the Death

We developed 10 items to assess parental preparations for their own death and 29 to assess parental preparations for the independent living of people with mental illnesses after their parent’s death. We used a three-point Likert scale (0 = never, 1 = conducted, 2 = conducted many times).

#### 2.3.5. Independent Living of Children with Mental Illness

We asked parents whether their children with mental illness had ever lived away from them without hospitalization, using a three-point Likert scale [0 (never), 1 (temporally lived), and 2 (independently living)].

### 2.4. Data Analysis

Data were analyzed using SPSS for Windows version 25 (IBM Corp, Armonk, NY, USA). We divided people with mental illnesses into three groups according to their experience of independent living away from their parents. Intergroup significant differences were indicated by *p*-values <0.05. We conducted the Mann–Whitney U test to verify whether there was a difference in the size of the ordinal scale data between the groups. We calculated Spearman’s rank correlation coefficient for correlations between ordinal scales and then tested for statistical significance with a test of no correlation. Logistic regression was used to analyze whether people with mental illnesses could live independently. We categorized the variables of independent living of people with mental illnesses as follows: 0 (never) was used as 0, and 1 (temporally lived) and 2 (independently living) were set as 1. To investigate multicollinearity between the independent variables, we assessed the variance inflation factor. None of the resulting variance inflation factors were less than 2. Therefore, multicollinearity was unlikely.

## 3. Results

Of the 1112 members, 447 returned questionnaires (response rate: 40.2%). We analyzed 357 respondents (fathers: *n* = 89, mothers: *n* = 268), excluding those with missing values and siblings (valid response rate: 32.1%).

### 3.1. Demographic Characteristics of the Participants

Table 1 presents the characteristics of people with mental illnesses and their parents. The age of participants with mental illnesses was the 40s, and that of their parents was the 70s. Living away from parents among people with mental illnesses was associated with their age and parental participation in the family support group. In contrast, low–level IADL were negatively associated with an individual’s independence from their parents.

Additionally, independent management of medications, no cohabitant, living at a rental house or group home, using social resources, obtaining welfare benefits, and meeting their partner was significantly associated with a person with mental illness’ independent living from their parents. In contrast, living with parents, maternal support in daily life, no income or pension for a disabled person, staying at home during free time, and parental livelihood being the same as the person with mental illness were significantly negatively associated with living with their parents.

### 3.2. Parental Preparations for Their Death and the Independent Living of Their Children with Mental Illness after the Death

Table 2 shows parental death preparations for their own death and for the independent living of their children with mental illness after the death. Four parental death preparations, including “proceeding to organize your own personal affairs”, “discussing the management and sale of the house with relatives”, “sharing management of the graves with the person with mental illness”, “preparing documents regarding advanced care plans in case of sickness” were significantly associated with living away from parents.

Additionally, 12 items including “gathering information on systems available to help independent living of the person with mental illness”, “families connecting with supporters and organizations”, “connecting with friends and neighbors who understand the person with mental illness”, “helping find supporters who are non-family members”, “consulting with family members about supporting the person’s life after parental death”, “discussing how to handle worsening medical conditions”, “ensuring housing for the person with mental illness”, “discussing what to do when the home requires repairs”, “creating a system for securing regular living expenses for the person with mental illness”, “calculating how much money is required for living expenses and sharing it with the person with mental illness”, “sharing how utilities and taxes will be paid with the person with mental illness”, “teaching methods of making do with a limited amount of money”, and “enabling the person with mental illness to conduct basic household chores” were associated with living away from parents.

### 3.3. Results of the Logistic Regression with the Dependent Variable for Whether or Not People with Mental Illness Can Live Independently

The results of the logistic regression analysis revealed the independent variables related to the independent living of people with mental illnesses (Table 3). The results showed that several factors were negatively associated with the independent living of people with mental illnesses, including maternal support in daily living (OR = 0.074, 95% CI = 0.018–0.297, *p* < 0.001), no income (OR = 0.031, 95% CI = 0.003–0.342, *p* = 0.005), receiving a pension for disabled people (OR = 0.042, 95% CI = 0.006–0.316, *p* = 0.002), staying at home during free time (OR = 0.031, 95% CI = 0.004–0.257, *p* = 0.001), and parental livelihood being the same as the person with mental illness (OR = 0.056, 95% CI = 0.011–0.282, *p* < 0.001). In contrast, parental participation in the family support group (OR = 3.349, 95% CI = 1.351–8.299, *p* = 0.009) and creating a system for securing regular living expenses for the person with mental illness (OR = 3.473, 95% CI = 1.346–8.844, *p* = 0.009) were positively associated with the independent living of people with mental illnesses.

## 4. Discussion

This cross-sectional study showed that the mean age of the people with mental illnesses was the 40s, and that of their parents was the 70s. Parents (in their 80s) support their adult children (in their 50s) with mental illness; the national government named this issue “the 8050 issue” [25]. The participants of this study were likely to face this issue. In Japan, the development of mental health services lags behind that of other developed countries [6,7]. Parents have addressed the shortage of social resources for mental disabilities and often feel the burden of providing care for their children [9,10,12]. To prevent the 8050 issue [28], family members should consider the recovery and independent life of their children with mental illnesses. Additionally, the findings suggest that community health and welfare staff need to urgently find and support these families before they face the limits of living in the community. Furthermore, local governments need to develop community mental health services to meet the needs and support the lives of people with mental illnesses, reduce stigma and discrimination against them, and promote the socialization of their care from their parents.

The results showed that mothers’ daily life support for their adult children with mental illnesses was negatively associated with their independent lives. Parents believe they need to be responsible for caring for their children for the rest of their lives [10]. Mothers are the main caregivers [29], which has continued during the COVID-19 pandemic [30]. Additionally, women have traditionally been expected to provide childcare and conduct house chores based on gender role expectations and social norms in Japan [31]. Therefore, mothers’ conduct of house chores and provision of care for people with mental illnesses can lead to difficulties in acquiring life skills for independent living of people with mental illnesses. Health and welfare staff should assess the extent to which mothers care for their children with mental illness and their life skills to live independently and recover.

We found that staying at home during free time was negatively associated with an individual’s independent life. Social exclusion and isolation were also common issues for people with mental illnesses with small social networks [32]. People with mental illnesses often only have contact with family members and healthcare staff [33]. Peer support services enhance patients’ recovery by sharing their life experiences [34]. Narratives help them recover in the community and discover new meanings in their lives [35]. Therefore, people with mental illnesses, their parents, and healthcare staff must learn from peer supporters how to use their free time to promote recovery in community settings.

The results showed that financial dependence on parents and poor income were negatively associated with independent living for people with mental illnesses. By contrast, creating a system for securing regular living expenses was positively associated with independent living. Poverty is a social determinant of health and the main issue for people with mental illnesses [36]. Owing to financial issues, they struggle to meet their basic needs, social exclusion, and deprivation [37]. Financial wellness is crucial to improve their financial capabilities and income to live in the community [38]. To promote their independent living in community settings, parents and healthcare staff should focus on the strengths and recovery processes of people with mental illnesses while improving their poverty and social barriers [39]. Our findings suggest that parents and healthcare providers of people with mental illnesses should collaborate with each other to enhance the financial basis for their children to live independently in the community. Parents need to plan how their children can secure their own living expenses without using parental pensions, salaries, and savings.

We found that parental participation in the family support group was positively associated with the independent living of adult children with mental illness. Caregivers of people with mental illnesses experience self-stigma [40], guilt, and shame [41]. Additionally, they feel burdens and distress and require peer support [42]. Gaining social support is crucial for improving the resilience of their families, responding to difficulties in providing daily care, and enhancing the recovery of their children [43]. Our findings suggest that parental participation in family support groups contributed to gaining social support and experimental knowledge from other parents whose children had already lived independently in community settings.

### Limitations and Future Research

This study had several limitations. First, because of the cross-sectional design of the study, we could not identify a cause-and-effect relationship. The respondents who were members of family association groups in Tokyo had a great interest in the theme of this study. However, the response rate was low. Therefore, there are limits to the generalizability of the findings. Second, the parents’ answers may not represent the actual situation of their children. Additionally, there could also be a recall bias in the responses of the older parents owing to their advanced age. Therefore, we carefully interpreted the results of the present study. Despite these limitations, the findings would be helpful for parents who live with their children with mental illness to forecast and enhance their preparation from an early stage of onset regarding the independent living of their children in the community to promote their recovery. In the future, we need to develop an educational program for parents of children with mental illnesses regarding their death and to promote their children’s independent living in the community.

## 5. Conclusions

This cross-sectional study examined the association between parental preparations for their death and the independent life and social participation of their children with mental illnesses. Logistic regression analysis showed that mothers’ support in daily life, staying at home during free time, and financial dependence on parents was negatively associated with the independent lives of adult children with mental illnesses. In contrast, creating a system for securing regular living expenses and parental participation in family groups were positively associated with such independent living. The results suggest that parents should enhance their children’s recovery and finances by forecasting their independent lives after their own death.

## Figures and Tables

**Table 1:** Characteristics of people with mental illnesses and their parents (*N* = 357).

Category	Variables		Never (*n* = 177)		Temporally Lived (*n* = 80)		Independently Living (*n* = 100)		Spearman’s Rank Correlation Coefficient	*p*-Value
			Mean	SD	Mean	SD	Mean	SD		
Characteristics of people with mental illnesses	Age		42.2	9.2	47.4	9.0	44.9	7.4	0.179	0.001
	Educational background		5.5	2.9	6.5	2.8	5.7	3.0	0.053	0.320
	Number of years since the onset of mental illness		20.4	6.1	23.9	7.6	20.1	5.5	0.033	0.547
	IADL: Preparation meals		2.1	1.1	1.8	1.2	0.9	1.3	−0.402	<0.001
	Housing chores		1.6	1.1	1.5	1.1	0.8	1.0	−0.313	<0.001
	Financial management		1.5	1.4	1.1	1.1	0.8	0.9	−0.249	<0.001
	Taking medication		0.6	1.0	0.4	0.9	0.3	0.7	−0.115	0.036
	Making telephone calls		0.5	1.1	0.4	1.0	0.1	0.5	−0.211	<0.001
	Shopping for groceries		1.0	1.1	0.5	0.7	0.3	0.6	−0.311	<0.001
	Using public transportation		0.7	1.1	0.4	0.9	0.2	0.7	−0.209	<0.001
Characteristics of parents	Age		72.1	8.8	75.4	7.9	74.5	7.6	0.149	0.005
	Educational background		6.1	2.8	6.5	2.8	6.5	2.7	0.067	0.209
	Household income		2.2	1.0	2.3	0.9	2.1	0.9	−0.014	0.792
	Participation in the family support group		1.2	0.8	1.4	0.7	1.4	0.7	0.166	0.002
	Family stigma		2.3	1.1	2.3	1.1	2.0	1.1	−0.091	0.090
**Category**	**Variables**		**Never** **(*n* = 177)** ***n***		**Temporally lived (*n* = 80)** ***n***		**Independently Living (*n* = 100)** ***n***		**Total**	***p*-Value**
Characteristics of people with mental illnesses	Sex	Male	113		52		59		224	0.540
		Female	61		27		39		127	
	Diagnosis	Schizophrenia	149		66		82		297	0.734
		Others	24		11		15		50	
	Independent management of medications	Possible	129		62		82		273	0.006
		Impossible	34		10		7		51	
	Cohabitant	None	1		1		65		67	<0.001
		Anyone	176		77		24		277	
		Parents	171		74		1		246	<0.001
		Others	6		4		88		98	
	Living place	Home	135		65		12		212	<0.001
		Others	42		15		87		144	
		Rental house	11		2		52		65	<0.001
		Others	166		78		47		291	
		Public housing	13		5		9		27	0.691
		Others	164		75		90		329	
		Group home	0		0		17		17	<0.001
		Others	177		80		82		339	
	Support in daily living	None	56		31		32		119	0.701
	(Multiple answers)	Anyone	120		49		66		235	
		By father	31		17		8		56	0.097
		Others	145		63		90		298	
		By mother	115		42		26		183	<0.001
		Others	61		38		72		171	
		Using social resources	33		17		48		98	<0.001
		None	143		63		50		256	
	Income	None	30		9		8		47	0.029
	(Multiple answers)	With income	147		70		92		309	
		Work-related salary	50		25		39		114	0.075
		Others	127		54		61		242	
		Pension for disabled person	141		62		66		269	0.02
		Others	36		17		34		87	
		Welfare benefit	0		2		35		37	<0.001
		Others	177		77		65		319	
	How to spend their free time (Multiple answers)	Staying at home	144		65		69		278	0.037
		Others	30		15		28		73	
		Meeting their partner	0		3		3		6	0.028
		Others	174		77		94		345	
		Exercise	71		33		41		145	0.822
		Others	103		47		56		206	
Characteristics of parents	Relationships	Mother	133		60		75		268	0.977
		Others	44		20		25		89	
	Livelihood being the same as the person with mental illness	Yes	153		71		9		233	<0.001
		No	18		6		86		110	
	Presence of disease under treatment	Under treatment	55		28		28		111	0.743
		No	117		50		69		236	

SD = Standard Deviation; IADL = Instrumental activities of daily living; Mann–Whitney U test.

**Table 2:** Parental preparations for their death and the independent living of their children with mental illness after the death (*N* = 357).

Category		Variables	*n*	Spearman’s Rank Correlation Coefficient	*p*-Value ^1^
Parental death preparation	1	Talking about your own death in the future to prepare the person with mental illness.	348	0.008	0.886
	2	Deciding whom you will consult first in case something happens to you with the person with mental illness.	351	0.027	0.615
	3	Making a list of related people and organizations that the person with mental illness should contact after the parents’ death.	345	0.018	0.740
	4	Proceeding to organize your own personal affairs.	349	0.150	0.005
	5	Discussing the management and sale of the house with relatives.	342	0.163	0.003
	6	Proceeding with the process of inheritance and division of property.	342	0.053	0.327
	7	Making a reservation for your own funeral.	345	0.026	0.626
	8	Sharing funeral information with the person with mental illness.	346	0.014	0.801
	9	Sharing management of the graves with the person with mental illness.	346	0.148	0.006
	10	Preparing documents regarding advanced care plans in case of sickness.	350	0.129	0.016
Parental preparation for independent living of the persons with mental illness in the community after their parental death	1	Gathering information on systems available to help independent living of the person with mental illness.	343	0.158	0.003
	2	Families connecting with supporters and organizations.	345	0.125	0.021
	3	Connecting with friends and neighbors who understand the person with mental illness.	337	0.145	0.008
	4	Helping find supporters who are non-family members.	339	0.144	0.008
	5	Discussing the relationship of the neighborhood.	339	0.066	0.224
	6	Discussing the dues and role of the neighborhood association.	339	0.073	0.179
	7	Discuss the rules for living in the community.	333	0.106	0.054
	8	Consulting with family members about supporting the person’s life after parental death.	334	0.114	0.038
	9	Discussing how to deal with their relatives.	339	0.007	0.903
	10	Discussing how to handle worsening medical conditions.	333	0.191	<0.001
	11	Support the management of their own medication and regular psychiatrist visits.	336	0.025	0.650
	12	Ensuring housing for the person with mental illness.	333	0.319	<0.001
	13	Discussing what to do when the home requires repairs.	330	0.203	<0.001
	14	Creating a system for securing regular living expenses of the person with mental illness.	335	0.272	<0.001
	15	Using systems in which the person’s money is used for their benefit.	336	0.079	0.151
	16	Sharing financial prospects after parental death with the person with mental illness.	334	0.073	0.185
	17	Calculating how much money is required for living expenses and sharing it with the person with mental illness.	336	0.266	<0.001
	18	Sharing how utilities and taxes will be paid with the person with mental illness.	337	0.295	<0.001
	19	Teaching methods of making do with a limited amount of money.	337	0.247	<0.001
	20	Sharing where bank books and seals are kept with the person with mental illness.	336	0.101	0.066
	21	Providing knowledge to the person with mental illness on avoiding scams.	337	0.093	0.089
	22	Enabling the person with mental illness to conduct basic household chores.	335	0.163	0.003
	23	Advise on avoiding chores they are not good at and utilizing other alternatives.	333	0.101	0.066
	24	Teaching the person with mental illness to separate and dispose of their garbage.	337	0.032	0.562
	25	Involving the person with mental illness to have fun in their lives.	343	−0.039	0.467
	26	Encouraging the person with mental illness to participate in society.	339	0.042	0.445
	27	Advising the person with mental illness on socializing.	337	0.084	0.122
	28	Encouraging the person with mental illness to work.	334	0.072	0.190
	29	Discussing what to do in the event of a disaster with the person with mental illness.	338	−0.060	0.270

^1^ Test for no correlation.

**Table 3:** Results of the logistic regression with the dependent variable for whether or not people with mental illness can live independently (*N* = 117).

Category	Variables	Items				95% CI		
			Estimate	SE	OR	Lower	Upper	*p*-Value
Characteristics of people with mental illnesses	Support for daily living: by mother (ref: No)	Yes	−2.60	0.71	0.074	0.018	0.297	<0.001
	Income (ref: Income such as salary)	None	−3.49	1.23	0.031	0.003	0.342	0.005
		Pension for disabled people	−3.16	1.03	0.042	0.006	0.316	0.002
	How to spend leisure time: stay home (ref: No)	Yes	−3.48	1.09	0.031	0.004	0.257	0.001
Characteristics of the parents	Livelihood being the same as that of the person with mental illness (ref: No)	Yes	−2.88	0.82	0.056	0.011	0.282	<0.001
	Participation in the family support group (ref: No)	Yes	1.21	0.46	3.349	1.351	8.299	0.009
	Creating a system for securing regular living expenses for the person with mental illness (ref: No)	Yes	1.25	0.48	3.473	1.364	8.844	0.009
	Constant		7.11	1.86	1226.00	-	-	<0.001

SE = standard error; OR = odds ratio; CI = confidence interval. Nagelkerke R^2^ = 0.703; Hosmer-Lemeshow test = 0.621.

## Data Availability

The data of this study are not publicly available to protect the participants’ privacy.
